# Multi-Mode GF-3 Satellite Image Geometric Accuracy Verification Using the RPC Model

**DOI:** 10.3390/s17092005

**Published:** 2017-09-01

**Authors:** Taoyang Wang, Guo Zhang, Lei Yu, Ruishan Zhao, Mingjun Deng, Kai Xu

**Affiliations:** 1 School of Remote Sensing and Information Engineering, Wuhan University, Wuhan 430079, China; wangtaoyang@whu.edu.cn (T.W.); yl121@whu.edu.cn (L.Y.); dmj2008@whu.edu.cn (M.D.); 2 State Key Laboratory of Information Engineering in Surveying, Mapping and Remote Sensing, Wuhan University, Wuhan 430079, China; 2015206190081@whu.edu.cn; 3 School of Geomatics, Liaoning Technical University, Fuxin 123000, China; zhaoruishan333@163.com

**Keywords:** GF-3, RPC model, geometric correction, accuracy

## Abstract

The GaoFen-3 (GF-3) satellite is the first C-band multi-polarization synthetic aperture radar (SAR) imaging satellite with a resolution up to 1 m in China. It is also the only SAR satellite of the High-Resolution Earth Observation System designed for civilian use. There are 12 different imaging models to meet the needs of different industry users. However, to use SAR satellite images for related applications, they must possess high geometric accuracy. In order to verify the geometric accuracy achieved by the different modes of GF-3 images, we analyze the SAR geometric error source and perform geometric correction tests based on the RPC model with and without ground control points (GCPs) for five imaging modes. These include the spotlight (SL), ultra-fine strip (UFS), Fine Strip I (FSI), Full polarized Strip I (QPSI), and standard strip (SS) modes. Experimental results show that the check point residuals are large and consistent without GCPs, but the root mean square error of the independent checkpoints for the case of four corner control points is better than 1.5 pixels, achieving a similar level of geometric positioning accuracy to that of international satellites. We conclude that the GF-3 satellite can be used for high-accuracy geometric processing and related industry applications.

## 1. Introduction

The GF-3 satellite is the only civilian microwave, remote-sensing, imaging satellite in the National High-Resolution Earth Observation System Major Project in China, and the first C-band and multi-polarization synthetic aperture radar (SAR) satellite [1]. The GF-3 satellite has 12 imaging modes, the most for any SAR satellite in the world [2]. It not only covers the traditional strip imaging mode and the scanning imaging mode, but also the Spotlight, strip, scan, wave, global observation, high and low incidence angle, and other imaging modes to achieve free switching spatial resolution ranges from 1 m to 500 m, and width ranges from 10 km to 650 km [3]. Specific parameters are shown in Table 1. The GF-3 satellite can not only perform wide range surveys, but also detailed investigation of specific areas, both of which can probe the ground and sea to achieve the “one-star multi-purpose” effect. The satellite has the ability to monitor global ocean and land resources in all weather situations and at all times of day. Use of the left and right attitude can expand the observation range and improve its rapid response ability. It can meet the needs of customers in the marine, disaster management, water conservation, and meteorology industries, among others [1].

Spaceborne SAR sensors are being continuously developed globally. With the recent launch of seven high-resolution SAR satellites, i.e., TerraSAR-X, COSMO-SkyMed, RADARSAT-2 (all in 2007), TanDEMX in 2010, KOMPSAT-5 in 2013, ALOS-2 in 2014, and Sentinel-1A in 2014, the geometric processing accuracy of SAR images has also advanced to the meter level, enabling images to be used for mapping large-scale features.

The GF-3 satellite, similar to the RADARSAT-2 satellite and TerraSAR-X satellite, can work in multiple modes and switch between 12 specific work modes (spotlight, strip, scanning, global observation, wave patterns, extended incidence angle mode, etc.) according to the bandwidth and spatial resolution requirements. The echo can also be received using a multi-polarization channel, thereby achieving multi-polarization observations. The observation patterns of the GF-3 satellite are shown in Table 1.

In different working modes, the GF-3 satellite has different geometric processing modes for different applications, and it is necessary to find a unified expression for multi-mode imaging data to simplify data processing and improve application efficiency.

In this study, we systematically analyze the combined characteristics of the GF-3 satellite system and the factors influencing satellite positioning. The common sensor, geometry-processing model RPC is used, and affine image transformation is used as the system compensation model for the geometric positioning error. The ground control points (GCPs) are obtained from a high-precision ground control source, and the geometric positioning accuracy of the SAR satellite image data is verified. Experimental results show that the check point residuals are large and consistent without GCPs, but the root mean square (RMS) error of the independent check points for the case of four corner control points is better than 1.5 pixels, achieving a similar level of geometric positioning accuracy to that of international satellites. The results of this study ensure that SAR images can be provided with high-precision positioning accuracy, thereby building a foundation for GF-3 satellite image applications in industry.

## 2. Error Sources of GF-3 Geometric Processing

The location of an arbitrary pixel in the SAR image is determined using the intersection of the centroid of the radar beam with the Earth’s surface. This intersection is determined by three fundamental relationships: a model describing the Earth's shape; the SAR Doppler equation defining the plane of the centroid; and the SAR range equation defining the distance from the sensor to the target [4]. Therefore, the processing accuracy of the target depends mainly on the ephemeris error of the satellite platform, the Doppler center frequency error, the azimuthal time error, the slant range measurement error, and the topographic error.

### 2.1. Ephemeris Error of the Satellite Platform

The error of the satellite position vector and the velocity vector can be separated into along-track directions, cross-track directions, and radial positions. Errors in the along-track directions result in an azimuthal error of the target position, which is almost equal to the orbit position error in the along-track directions. Errors in the cross-track directions mainly lead to a range error of the target position, while the effect of the Earth’s rotation on the target azimuth position can be ignored. Errors in radial positions can also lead to a range error of the target position, which is due to the azimuth offset of the Doppler center frequency. The error of the satellite velocity vector can also be separated in the above three directions, which results in offset of the Doppler center frequency and the azimuthal error of the target position. The range position error caused by the sensor velocity error can be ignored; however, the lateral velocity error will produce an azimuth proportional error in the image [5].

### 2.2. Doppler Center Frequency Error

According to the Doppler equation, the azimuthal position of the ground pixel is determined by the Doppler center frequency of the pixel. If the Doppler center frequency used in azimuthal compression is not consistent with the actual Doppler center frequency, it will cause displacement of the ground target in the azimuthal direction. Currently, the estimation error of Doppler parameters can be controlled within 3 Hz through clutter lock technology and self-focusing technology [6]. Therefore, the azimuthal error, due to the Doppler center frequency error, can be ignored.

### 2.3. Azimuthal Time Error

As it is impossible for satellites to provide real-time ephemeris data, and the corresponding satellite ephemeris can be obtained only by means of orbit interpolation, the main factors affecting the azimuthal time of the satellites include the RMS of PRF (pulse repetition frequency) caused by the RMS of STALO (stable local oscillator frequency), and the loss of image lines. GF-3 designed the second pulse time mechanism, counting each pulse to obtain an accurate azimuth time, the accuracy of which can reach 30 ns. The azimuthal error brought by this method, which is approximately 0.2 mm, can be ignored [7].

### 2.4. Slant Range Measurement Error

The slant range measurement error will cause the field of view of the target to move along the equal Doppler line, which can result in a positioning error. The accuracy of the slant range measurement depends on the time delay, which is at the speed of light of the target echo received by SAR with respect to the transmitted pulse. There are three types of time delay of the slant range measurement in direct images of the digital acquisition system, which include the start delay time of the echo sampling window relative to the pulse emission time, the electronic delay from pulse emission to acceptance, and the data sampling time delay caused by the range compression algorithm in signal processing. Range pulse compression is performed by correlating the backscattered dispersed pulses from the imaging surface with a replica of the transmitted signal [8]. The start delay error of the echo sampling window is the main error source, while the effects of the other two errors are less important. Therefore, the main reason for a slant range measurement error is inaccuracy in estimating the system delay time.

### 2.5. Topographic Error

**Figure 1:**
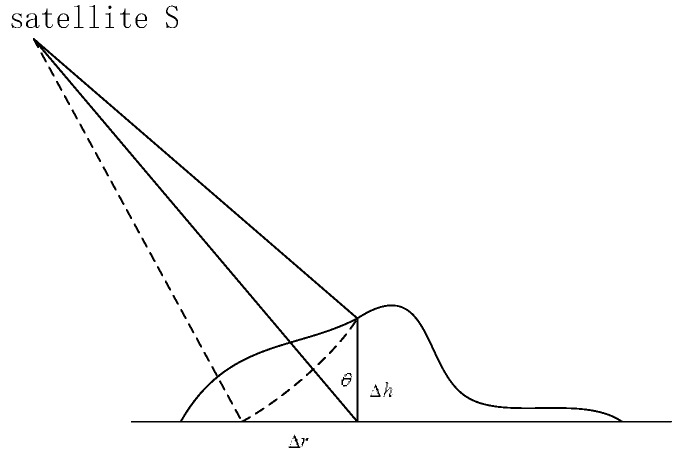
Positioning error caused by topographic relief.

The surface of the Earth rises and falls everywhere, and any Earth model is only an approximate description of the actual Earth; thus, errors are bound to exist. As the SAR system is slant range imaging and receives the backscattered signals of ground objects, topographic relief will affect the quality of SAR images and the accuracy of geometric processing. When estimating the height of the target, the topographic error can be represented by the effective slant range error. As shown in Figure 1, a ground elevation of ∆h will result in a slant range error of ∆R and a horizontal range error of ∆r. This can be approximated as:

(1) $$\Delta r=\frac{\Delta h}{\tan\;\theta }$$

where θ is the incident angle. When ∆h is constant, a smaller incident angle will result in a greater positioning error. Therefore, a high precision DEM is used as elevation data in this study.

## 3. General Geometric Processing Model of GF-3

The Range-Doppler (RD) model is generally adopted to deal with the geometric processing of SAR images. However, this takes a great deal of time as both the direct and inverse transformations of the RD model are iterative processes [9,10]. Moreover, the RD model is dependent on sensors and platforms (different spaceborne SAR images have different auxiliary data and image formats); thus, it is necessary to find a new method to replace the RD methodology.

The RPC model is a generalized sensor model used as an alternative to the rigorous sensor model (RSM). The RPC model makes full use of the auxiliary parameters of satellite images to create a general model that can then be fitted to an RSM to solve for its coefficients.

The RPC model establishes the relationship between ground coordinates (latitude, longitude, height) and corresponding pixel coordinates (line, sample). To improve the numerical stability, 2D image coordinates and 3D ground coordinates are offset and scaled to within the range of −1.0 to 1.0. The RPC model can be defined as follows [11]:

(2) $$r=\frac{Num_{L}\left(X,Y,Z\right)}{Den_{L}\left(X,Y,Z\right)};\;c=\frac{Num_{S}\left(X,Y,Z\right)}{Den_{S}\left(X,Y,Z\right)}$$

where X is the normalized latitude, Y is the normalized longitude, and Z is the normalized height. r is the normalized line number, c is the normalized sample number, and $Num_{L}\left(X,Y,Z\right)$, $Den_{L}\left(X,Y,Z\right)$, $Num_{S}\left(X,Y,Z\right)$, and $Den_{S}\left(X,Y,Z\right)$ are the terms of the third-order polynomial of (X,Y,Z). For example, the form of the polynomial $Num_{L}\left(X,Y,Z\right)$ is:

(3) $$\begin{array}{cc} Num_{L}\left(X,Y,Z\right)= & a_{i0}+a_{i1}Z+a_{i2}Y+a_{i3}X+a_{i4}ZY+a_{i5}ZX+a_{i6}YX+a_{i7}Z^{2}+a_{i8}Y^{2}\\ & +a_{i9}X^{2}+a_{i10}ZYX+a_{i11}Z^{2}Y+a_{i12}Z^{2}X+a_{i13}Y^{2}Z+a_{i14}Y^{2}X\\ & +a_{i15}ZX^{2}+a_{i16}YX^{2}+a_{i17}Z^{3}+a_{i18}Y^{3}+a_{i19}X^{3} \end{array}$$

where *a_ij_* (*i* = 1, 2, 3, 4; *j* = 0, 1, …, 19) are rational polynomial coefficients (RPCs). There are 80 total parameters for the RPCs. To ensure the reliability of the calculation, the first parameter coefficient of the denominator term is often set to 1, so the 80 parameters for the RPCs turn to 78 parameters.

Although most research focusing on frame camera and/or push broom scanner imagery has used the RPC model to replace the RSM, the RPC model has rarely been applied to SAR image processing. Nonetheless, as has been discussed in detail in [12], the RPC model can be used as a replacement for the RD model, which is a conventional geometric SAR model. When the RD model is available, the parameters can always be solved in a terrain-independent manner [13]. The detailed process for a terrain-independent solution for the RPC model has been discussed in [14,15].

**Figure 2:**
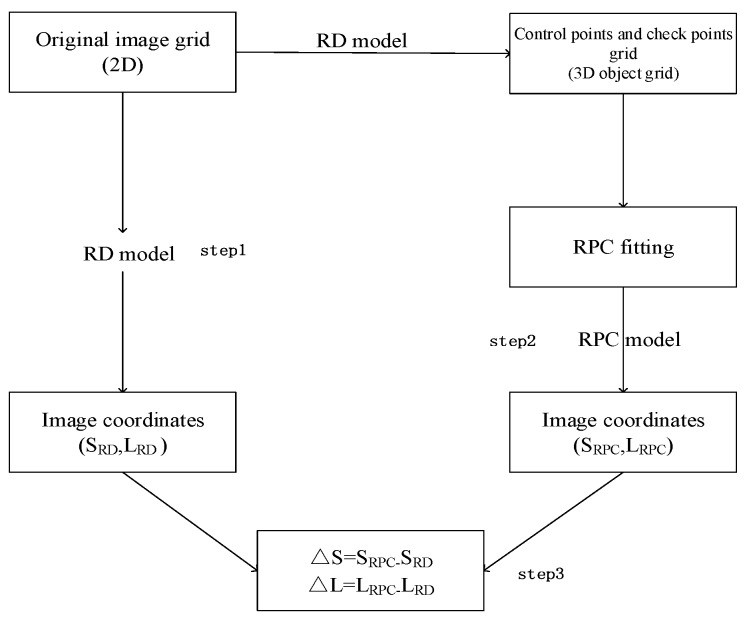
Flowchart of the rational polynomial coefficient (RPC) model solution process.

The proposed estimation process, using a least-squares approach, requires only the RD model and the maximum and minimum heights in the image area, which can be extracted from the global DEM supplied by the United States Geological Survey (USGS). As shown in Figure 2, this method involves three main steps [13,16]:

1. Determination of an image grid and establishment of a 3D object grid of points using the RD model;
2. RPC fitting;
3. Accuracy checking.

A model commonly used for system error compensation based on the image compensation scheme is the affine transformation model, which involves six unknowns and requires a minimum of three GCPs for calculating the parameters. The two offset parameters, e0 and f0, can be solved for using only one GCP, which can absorb most of the errors. The offset and drift parameters can be solved by using two GCPs simultaneously:

(4) $$\{\begin{array}{l} \Delta r=e_{0}+e_{1}r+e_{2}c\\ \Delta c=f_{0}+f_{1}r+f_{2}c \end{array}$$

where $\left(e_{0},e_{1},e_{2},f_{0},f_{1},f_{2}\right)$ are parameters of the affine transformation, (Δr,Δc) are values for compensation of systematic errors of the image point, and (r,c) are the coordinates of the image point [17,18,19].

Based on Equations (2) and (4), the affine transformation parameters $\left(e_{0},e_{1},e_{2}\right)$ and $\left(f_{0},f_{1},f_{2}\right)$ of the image space compensation should be set as unknowns. Based on the RFM, the image orientation error equation for ground control points is:

(5) $$\left[\begin{array}{c} v_{r}\\ v_{c} \end{array}\right]=\left[\begin{array}{cccccc} \frac{\partial r}{\partial e_{0}} & \frac{\partial r}{\partial e_{1}} & \frac{\partial r}{\partial e_{2}} & 0 & 0 & 0\\ 0 & 0 & 0 & \frac{\partial c}{\partial f_{0}} & \frac{\partial c}{\partial f_{1}} & \frac{\partial c}{\partial f_{2}} \end{array}\right]\cdot \left[\begin{array}{c} \Delta e_{0}\\ \Delta e_{1}\\ \Delta e_{2}\\ \Delta f_{0}\\ \Delta f_{1}\\ \Delta f_{2} \end{array}\right]-\left[\begin{array}{c} r-\hat{r}\\ c-\hat{c} \end{array}\right]$$

Also, this error equation can be solved according to the principle of least-squares adjustment. Finally, the orientation accuracy of GF-3 is evaluated by the RMS errors of the check points.

## 4. Experimental Data

In this study, SAR slant range images of GF-3 were used as test data. To validate the geometric accuracy of the orthophoto, experimental data was selected from three scientific test fields in China, Mount Song, Tianjin, and Taiyuan respectively. A total of five types of GF-3 L1A level data were used, including images of SLC and RPC model files, and including spotlight (SL, 1 m resolution), ultra-fine strip (UFS, 3 m resolution), fine strip (FSI, 5 m resolution), full polarization strip (QPSI, 8 m resolution), and standard strip (SS, 25 m resolution). The five data modes are shown in Table 2.

Because ground objects in SAR images are harder to identify than features in optical images, the annual measurement accuracy of GCPs in both SAR images was approximately ±1 pixels. All the GCPs represented salient ground features such as road intersections or corners of water bodies. The GCPs in all test areas were obtained from the 1:5000 DOM and DEM, with a spatial accuracy of ± 0.1 m in plane and elevation.

Due to different imaging principles and image textures, it was difficult to extract corresponding image points from an SAR image and an optical image. Zhao et al. [20] offers the following selection tips:

1. Choose a crossroads intersection or a T-junction. These junctions have clear road textures in SAR images and are easy to identify in the corresponding optical image.
2. Select roads of an appropriate size. Roads that are too narrow are difficult to identify clearly, whilst roads that are too wide make it difficult to determine an accurate centerline position.
3. Choose straight roads. Due to speckle noise, a road boundary in SAR images is not as clear as it is in optical images, especially at intersections. If the roads are straight, the centerline at intersections can be accurately determined.
4. Select roads in a flat area. Undulating roads, together with circumjacent buildings, may lead to foreshortening, layover, or shadow, which affects the accuracy of the selected GCPs.

Following the tips above can ensure that the accuracy of GCPs which are measured on the image is about 1 pixels (by artificially comparing the same features of the ground objects on the optical and SAR images). If the measurement accuracy of GCPs in an image space is low, the gross error part of some GCPs will be allocated to other control points on the image through the least square adjustment, which leads to a decrease in the orientation accuracy of the whole image.

## 5. Experimental Results

Experiments with different types of SAR data were performed to verify the RPC parameters generation method. For high-resolution satellite SAR imagery, the accuracy is extremely high. For COSMO-SkyMed, RADARSAT-2, and TerraSAR-X data, the RMS and maximum error of model fitting is 10-4 pixels or less. The results show that RPC is fully capable of replacing the RD model [15,16,21]. Therefore, the RPC parameters of GF-3 used in this article are generated by the scheme.

First, under the ground control program with zero control points, four control points, and full control points, respectively, the GF-3 images of different regions were tested using a single image to evaluate the geometric positioning accuracy of the different modes.

**Figure 3:**
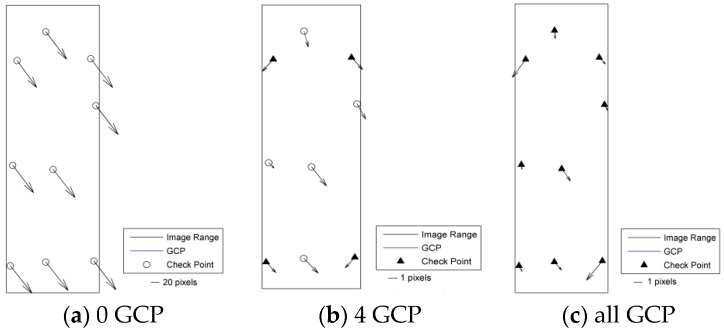
Residual distributions of check points of the SL mode orientation for Taiyuan. (**a**) 0 GCP; (**b**) 4 GCP; (**c**) all GCP.

**Figure 4:**
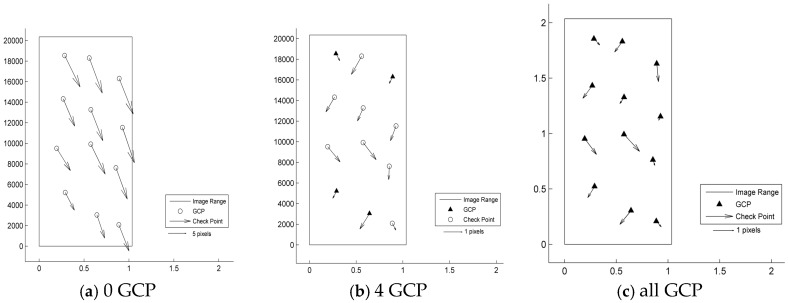
Residual distributions of check points of the UFS mode orientation for Tianjin: (**a**) 0 GCP; (**b**) 4 GCP; (**c**) all GCP.

**Figure 5:**
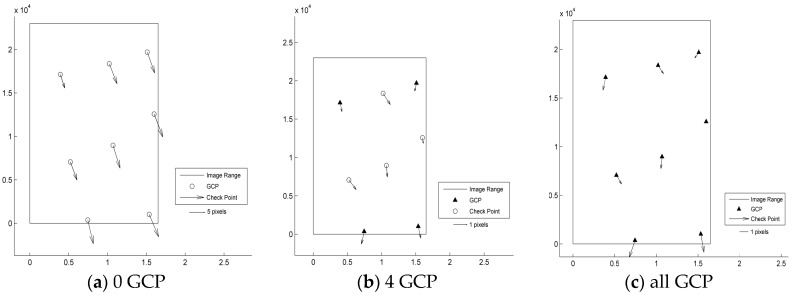
Residual distributions of check points of the FSI mode orientation for Mount Song: (**a**) 0 GCP; (**b**) 4 GCP; (**c**) all GCP.

**Figure 6:**
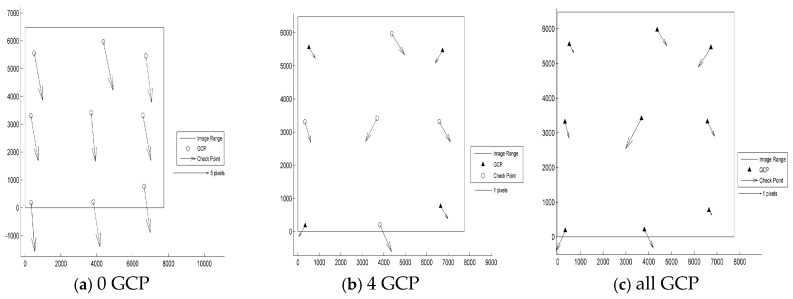
Residual distributions of check points of the QPSI mode orientation for Tianjin: (**a**) 0 GCP; (**b**) 4 GCP; (**c**) all GCP.

**Figure 7:**
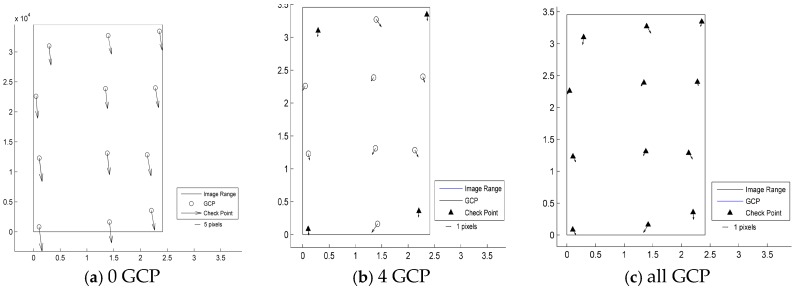
Residual distributions of check points of the SS mode orientation for Mount Song: (**a**) 0 GCP; (**b**) 4 GCP; (**c**) all GCP.

According to the results shown in Table 3 and Figure 3, Figure 4, Figure 5, Figure 6 and Figure 7, the image orientation results for zero control points show consistent residual distributions of checkpoints in both magnitude and direction. The error distribution law conforms to the affine change in the image space, and the affine model can be used to compensate for the system error.

When a control point is laid at each of the four corners, the system error is significantly reduced, and the residual error of the checkpoints is not consistent. After the errors are endowed with a reasonable value by least squares adjustment in the image space, all points converge to their probable position through iterations. According to the different patterns of image space statistics, the geometric positioning accuracy of GF-3 can reach 1.5 pixels. So, the image orientation scheme of four control points laid in the corners respectively for GF-3 is recommended. Finally, when all the control points are involved in the adjustment calculation, the limit precision of the directional approach is almost 1 pixel, indicating that the image accuracy of the control point itself is approximately 1 pixel.

Using DEM data of the control data source, orientation parameters are obtained for the case of four corner control points and for each ortho-rectification image. For the ortho-image, the check points are checked again to verify the geometric accuracy. The results are shown in Table 4. The single image ortho-rectification accuracy results and orientation precision are almost consistent, indicating that a high-precision DEM can effectively eliminate the differences caused by projection of the terrain. 

The accuracy of GF-3 was compared with that of equivalent satellites with similar payload types around the world, and the results are shown in Table 5. In terms of both resolution and geometric positioning accuracy, the GF-3 satellite compares well with other satellites such as TerraSAR-X, RADARSAT-2, and ERS.

## 6. Conclusions

In this study, we validated the geometric accuracy of slant range imagery from the GF-3 satellite after an on-orbit test period. We analyzed the error source of the GF-3 satellite image target, and used the general sensor model based on the RPC model to perform single image orientation and ortho-rectification for five modes of GF-3 1A level data. Experimental results have shown that the check point residuals are large and consistent without GCPs, but the root mean square (RMS) error of the independent checkpoints for the case of four corner control points is better than 1.5 pixels, therefore achieving a similar level of geometric positioning accuracy to that of other satellites, such as TerraSAR-X, COSMO, and RADARSAT-2.

The GF-3 can therefore be used for high-accuracy geometric processing. Furthermore, it is applicable in marine environment monitoring and protection, disaster monitoring and assessment of water conservancy facilities, monitoring and evaluation of water resources management, meteorology and other fields. It is an important technical support for land resources monitoring and emergency disaster prevention and mitigation implementation of ocean development.

## Figures and Tables

**Table 1:** Observation patterns of the GF-3 satellite.

No.	Work Modes		Incidence Angle (°)	Look Number	Resolution (m)			Imaging Bandwidth (km)		Polarization Mode
					Nominal	Azimuth	Range	Nominal	Size	
1	spotlight (SL)		20–50	1 × 1	1	1.0~1.5	0.9~2.5	10 × 10	10 × 10	Optional single polarization
2	ultra-fine strip (UFS)		20–50	1 × 1	3	3	2.5~5	30	30	Optional single polarization
3	fine strip I (FSI)		19–50	1 × 1	5	5	4~6	50	50	Optional dual polarization
4	fine strip II (FSII)		19–50	1 × 2	10	10	8~12	100	95~110	Optional dual polarization
5	standard strip (SS)		17–50	3 × 2	25	25	15~30	130	95~150	Optional dual polarization
6	narrow scan (NSC)		17–50	2 × 3	50	50~60	30~60	300	300	Optional dual polarization
7	wide scan (WSC)		17–50	2 × 4	100	100	50~110	500	500	Optional dual polarization
8	global (GLO)		17–53	4 × 2	500	500	350~700	650	650	Optional dual polarization
9	full polarized Strip I (QPSI)		20–41	1 × 1	8	8	6~9	30	20~35	Full polarization
10	full polarized Strip II (QPS II)		20–38	3 × 2	25	25	15~30	40	35~50	Full polarization
11	wave imaging (WAV)		20–41	1 × 2	10	10	8~12	5 × 5	5 × 5	Full polarization
12	extended (EXT)	low	10–20	3 × 2	25	25	15~30	130	120~150	Optional dual polarization
		high	50–60	3 × 2	25	25	20~30	80	70~90	Optional dual polarization

**Table 2:** Basic parameters of test area data for the five GF-3 SAR images over the study site.

Imaging Mode	Acquisition Date	Orbit	Image Size (Pixel)	Central Look Angle	Imaging Region
spotlight (SL)	2 March 2017	ASC	10861/33766	27.17	Taiyuan
ultra-fine strip (UFS)	24 February 2017	DEC	10352/20358	21.27	Tianjin
fine strip I (FSI)	30 December 2016	ASC	16509/23002	38.66	Mount Song
full polarization strip (QPSI)	30 March 2017	DEC	7750/6482	31.70	Tianjin
standard strip (SS)	26 January 2017	ASC	24131/34568	18.77	Mount Song

**Table 3:** Single image orientation accuracy of five GF-3 satellite image modes (pixel).

Image Mode	Test Site	GCP Number	Check Point Number	Root Mean Square Error (RMSE) of GCP (Pixels)			Root Mean Square Error (RMSE) of Checkpoint (Pixels)		
				x	y	Plane	x	y	Plane
SL	Taiyuan	0	9	-	-	-	42.4637	50.2833	65.8147
		4	5	0.7140	1.1387	1.3441	0.8867	1.1465	1.4494
		9	0	0.8578	0.9767	1.2999	-	-	-
UFS	Tianjin	0	11	-	-	-	12.5462	6.8061	14.2734
		4	7	0.5460	0.3933	0.6729	1.1250	0.9719	1.4867
		11	0	0.6871	0.7818	1.0408	-	-	-
FSI	Mount Song	0	8	-	-	-	10.9848	4.7005	11.9483
		4	4	1.3287	0.3376	1.3709	1.0355	0.7581	1.2834
		8	0	1.1153	0.4758	1.2126	-	-	-
QPSI	Tianjin	0	9	-	-	-	8.1886	1.9381	8.4148
		4	5	0.1465	0.3220	0.3538	0.4513	0.5124	0.6828
		9	0	0.3146	0.4059	0.5135	-	-	-
SS	Mount Song	0	12	-	-	-	17.2819	2.5586	17.4703
		4	8	0.9236	0.1070	0.9298	0.7041	0.6708	0.9725
		12	0	0.7463	0.5044	0.9008	-	-	-

**Table 4:** Table of ortho-rectification accuracy comparison among the five GF-3 satellite imaging modes.

Image Mode	Test Filed	RMSE of Checkpoint (Pixels)		
		DX	DY	Plane
SL	Taiyuan	1.1049	1.0061	1.4943
UFS	Tianjin	5.7351	4.9067	4.6129
FS1	Mount Song	5.1171	3.5901	6.2509
QPS1	Tianjin	5.1347	4.3998	6.7619
SS	Mount Song	14.1420	18.0254	22.9109

**Table 5:** Comparison between the accuracy (RMSE) of GF-3 and other satellites.

Item	TerraSAR-X [22]	COSMO-SkyMed [23]	RADARSAT-2 [24]	ERS [25]	GF-3
Country	Germany	Italy	Canada	Europe	China
Image resolution (m)	1/3	1/15	3/8	30	1-500
Geometric accuracy in ground space(m)	2	5	8	10	1.49
